# The HSP Immune Network in Cancer

**DOI:** 10.3389/fimmu.2021.796493

**Published:** 2021-11-30

**Authors:** Zarema Albakova, Yana Mangasarova

**Affiliations:** ^1^ Department of Immunology, Lomonosov Moscow State University, Moscow, Russia; ^2^ National Research Center for Hematology, Moscow, Russia

**Keywords:** heat shock proteins, tumor immunity, immunotherapy, cancer, extracellular HSPs

## Abstract

Heat shock proteins are molecular chaperones which support tumor development by regulating various cellular processes including unfolded protein response, mitochondrial bioenergetics, apoptosis, autophagy, necroptosis, lipid metabolism, angiogenesis, cancer cell stemness, epithelial-mesenchymal transition and tumor immunity. Apart from their intracellular activities, HSPs have also distinct extracellular functions. However, the role that HSP chaperones play in the regulation of immune responses inside and outside the cell is not yet clear. Herein, we explore the intracellular and extracellular immunologic functions of HSPs in cancer. A broader understanding of how HSPs modulate immune responses may provide critical insights for the development of effective immunotherapies.

## Introduction

Heat shock proteins (HSPs) are molecular chaperones classified into several families such as HSP70, HSP90, HSP110, HSPB, HSP40 and chaperonins (1). HSPs are induced upon various cellular stresses including heat, radiation, infectious agents, heavy metal toxicity and hypoxia (2). Recent data have emerged suggesting that HSP functions are not only dependent on the stimuli triggering their expression, but also the compartment in which they are present (3–9). HSPs have been implicated in the regulation of apoptosis, necroptosis, autophagy, cancer cell stemness, epithelial to mesenchymal transition, lipid metabolism, angiogenesis and tumor immunity, supporting tumor growth and development (10–14). Originally described as intracellular chaperones, HSPs have also been found in extracellular milieu. In extracellular environment, presence of HSPs associates with tumor aggressiveness, resistance to therapy and poor clinical outcome (15–17). Various HSPs have been detected in liquid biopsies of cancer patients, stimulating the research towards the discovery of HSP-based specific biomarker of cancer [reviewed in (18)] (19–32).

Increasing evidence has shown that extracellular HSPs are capable of triggering immune responses, which was further translated into the development of various HSP-based immunotherapies (33–39). Furthermore, several studies reported that different populations of immune cells including natural killer (NK) cells, T cells, monocyte-derived dendritic cells (mDCs), platelets and neutrophils may release HSPs in extracellular vesicles [reviewed in (18)] (40–46). This review will focus on immunologic functions of HSPs in tumor immunity. Further elucidating the role of HSPs in tumor immunology, may provide the basis for future discoveries of novel and effective HSP-based immunotherapies.

## Immune Functions of HSPs in Cancer

HSPs are localized in various cellular compartments including cytosol, endoplasmic reticulum (ER) and mitochondria, where they play compartment-specific cellular functions. For example, ER-resident HSP90 chaperone glucose-regulated protein 94 (GRP94, also known as Gp96) plays critical role in unfolded protein response (UPR) while mitochondria-resident HSP90 chaperone tumor necrosis factor receptor-associated protein 1 (TRAP1) is involved in mitochondrial bioenergetics, contributing to apoptosis resistance, cancer cell stemness and Warburg phenotype (47–50). Apart from their compartment-specific role, HSPs play an important role in the regulation of immune responses. Bae and colleagues reported that HSP90 inhibition reduces surface expression of CD3, CD4, CD8, CD28, CD40L, CD25 and αβ on T cells and activating receptors such as CD2, CD11a, CD94, NKp30, NKp44, NKp46, KARp50.3 on the surface of NK cells (51). HSP90 inhibition reduces NK and T cell proliferation, NK cell cytotoxic activity and IFN-γ production by T and NK cells (51, 52). By contrast, ER HSP90 member GRP94 does not affect cytolytic activity of NK cells towards tumors but rather acts indirectly *via* antigen presenting cells (APC) selectively enhancing cytokine production (53). Earlier studies showed that HSPs carry tumor-derived peptides that may induce T- cell mediated response while HSPs may stimulate NK cells in the absence of antigenic peptide (45, 46). In this regard, Multhoff and colleagues reported that pre-treatment of NK cells with stress-inducible HSP70 or HSP70-derived TKD peptide in combination with IL-2 or IL-15 induces NK cell proliferation and cytolytic activity (54, 55). NK cells pre-stimulated with IL-2 and TKD in combination with anti-PD-1 antibody improves cytolytic activity of NK cells against tumor cells and inhibits tumor growth *in vivo* (56). Notably, adoptive therapy with autologous NK cells *ex vivo* stimulated with TKD and IL-2 increased the number of activated NK cells in the blood of patients with membrane-positive HSP70 non-small cell lung carcinoma after radiochemotherapy in phase II clinical trial (36). Taken together, HSPs regulate the expression of critical antigens and co-stimulatory molecules on T cells and key activating receptors on NK cells while HSPs inhibition impairs proliferation and cytotoxic activity of T and NK cells, suggesting that HSPs are critical for the regulation of the phenotype and functional activity of T and NK cells.

HSP70s may also act as damage-associated molecular patterns (DAMPs) and elicit anti-tumor response which with long-term exposure results in immune tolerance (11, 57–59). Acting as a DAMP, HSP70 negatively regulates multimeric cytosolic protein complex - the Nod-like receptor protein 3 (NLRP3) inflammasome (60). NLRP3 is an intracellular sensor that detects endogenous danger signals, leading to the assembly of NLRP3 inflammasome, which further activates the caspase-1- dependent release of pro-inflammatory cytokines such as IL-1β and IL-18 (61). In this regard, HSP70 deficiency leads to the activation of caspase-1 and subsequent production of IL-1β by bone marrow-derived macrophages (60). Conversely, HSP90 downregulation inhibits priming and activation of NLRP3 inflammasome (62). HSP90 inhibition in macrophages showed to affect an inflammatory response to lipopolysaccharide and IFNγ, resulting in reduced secretion of IL-6, TNFα and nitric oxide (NO) (63). Since HSP90 stabilizes IKK complex, the expression of IKK was also diminished, leading to inactivation of NF-kB pathway (63–65). Therefore, the overexpression of HSP70 and HSP90 in the cytosol will either inhibit or activate NLRP3 inflammasome, respectively, suggesting that functions of immune cells that express NLPR3 inflammasome (e.g. macrophages, monocytes and CD4+T cells) may be affected by HSPs up- or down-regulation. For example, inflammasome activation in CD4+T cells leads to increased IFNγ secretion and T helper (Th)1 differentiation, and, therefore, various HSP90 and HSP70 inhibitors may differently affect Th1 response, however, this warrants further investigation (66).

HSPs such as stress-inducible cytosolic HSP90 family member HSP90α (HSP90AA1) and constitutive cytosolic HSP90 member HSP90β (HSP90AB1) also play an important role in antigen presentation. Ichiyanagi and co-workers demonstrated that heat shock factor -1 (HSF-1)-deficient DCs are less efficient in cross-presentation of antigens (67). Furthermore, HSP90α-deficient DCs showed reduction in cytosolic translocation of antigens (65, 68). Along this line, Kunisawa and colleagues reported that, in contrast to HSP90β, specific inactivation of HSP90α leads to a loss of proteolytic intermediates and reduced presentation of the final peptide on the cell surface of MHC I molecule (69). Besides MHC class I antigen presentation, HSP90 inhibition also downregulates MHC II-mediated presentation of endogenous and exogenous peptides by APC (70). Additionally, constitutive HSP70 family member HSC70 is also involved in MHC class II antigen presentation pathway (71, 72). HSC70 interacts with MHC II for delivering clients to lysosomes (73). Cumulatively, these studies suggest a major role of HSPs in MHC I and MHC II antigen presentation, suggesting that HSP inhibition may affect immune responses in various treatment scenarios.

HSPs are also implicated in the regulation of immune checkpoints. Song and colleagues reported that HSP90α inhibition sensitizes tumor cells to anti-PD-1 blockade (74). In a recent study, Zavareh and co-workers (2021) demonstrated that HSP90 inhibition by ganetespib reduces surface expression of PD-L1 on MC-38 tumor cells and human monocyte-derived macrophages (75). Mechanistically, HSP90 inhibition downregulates c-MYC and signal transducer and activator of transcription 3 (STAT3), leading to the reduction of PD-L1 surface expression (75). In this regard, Marzec and colleagues demonstrated that HSP90 client protein nucleophosmin/anaplastic lymphoma kinase (NPM/ALK) induced PD-L1 surface expression *via* the activation of STAT3 in T cell lymphoma (76). Since c-MYC and NPM/ALK are HSP90 client proteins, it appears that HSP90 inhibition downregulates PD-L1 surface expression *via* the degradation of HSP90 client proteins (c-MYC and NPM/ALK). Notably, anti-PD-L1 in combination with HSP90 inhibitor ganetespib showed higher anti-tumor activity than anti-PD-L1 alone in syngeneic mouse models (77). Furthemore, ganetespib in combination with anti-PD-L1 showed to increase the number of activated CD8+ T cells (75). Earlier, Mbofung and colleagues (2017) demonstrated that mice treated with ganetespib and anti-CTLA4 increased the number of CD8+T cells while decreasing the number of T regulatory cells (78).Furthermore, ganetespib upregulated interferon response genes, sensitizing human melanoma cells to T-cell mediated killing (78). D’Arrigo and colleagues reported that downregulation of the spliced form of HSP90 cochaperone FKBP51 (sFKBP51) reduces PD-L1 expression in glioma cells (79). In another study, HSP70 ER member glucose-regulated protein 78 (GRP78) downregulation decreased PD-L1 expression in breast cancer cells (80). Taken together, HSPs regulate the expression of multiple immune checkpoints including PD-L1 and PD-L2 while combination of anti-PD-L1, anti-PD-1, anti-CTLA4 with HSP90 inhibitor showed promising results in mouse models, suggesting that HSP inhibitors may further improve immunotherapy.

## Extracellular HSPs and Tumor Immunity

In extracellular environment HSPs exist in several forms either secreted or membrane-bound. In this regard, Multhoff and colleagues demonstrated that surface expression of HSP70 on tumors does not involve classical ER-Golgi transport pathway for its membrane localization (81). Mambula and Calderwood reported that HSP70 can be released *via* lysosomal endosomes (82, 83). Authors also showed that HSP70 release involves the entry of HSP70 into endolysosomes *via* ATP-binding cassette (ABC) transporters (82, 83). HSP70 may also interact with either globotriaoslyceramide or phosphatidylserine for the anchorage of HSP70 in the plasma membranes of tumors (84, 85). Another mechanism by which HSPs are secreted into extracellular milieu involves the release of exosomes derived from multivesicular bodies (86–89). In this regard, several studies reported that HSPs on the surface of tumor-derived exosomes promote tumor growth by suppressing immune responses (90, 91).

### Extracellular HSP90

Ullrich and co-workers (1986) reported the expression of tumor-specific transplantation antigen on the surface of tumor cells which they identified as HSP90 (92). Immunization of mice with this antigen inhibited tumor growth, suggesting that extracellular HSP90 (eHSP90) contributes to anti-tumor immunity (92, 93). Hostile tumor microenvironment leads to chronic ER stress, resulting in the elevation of extracellular HSPs. In this regard, Tramentozzi and colleagues observed high expression of extracellular GRP94-IgG complexes in the plasma of cancer patients (94). GRP94 alone or bound to IgG promotes angiogenesis, MMP-9 expression and extracellular release of HSP90α and HSP70 in human umbilical vein endothelial cells (HUVECs) (94–96). Authors showed that GRP94 alone may promote angiogenic transformation *via* stimulation of ERK1/ERK2 pathway (96). eGRP94 also induces maturation of mDCs, increasing surface expression of CD86 and CD83 (97). GRP94-treated mDCs strongly induces T cell proliferation (97). Interaction of CD91 with GRP94 leads to increased secretion of several inflammatory cytokines such as IL-1β, IL-6 and TNF-α by RAW264.7 cells (98). Dai and colleagues reported that GRP94 on the surface of tumor cells also induces CD4+ and CD8+ T cell memory response (99). eGRP94 facilitates cross-presentation of MHC class I and elicits CD8+ T cell response (100). Even though GRP94 facilitates the presentation of MHC class II-restricted peptides, CD4+T cells are not capable of secreting Th1 and Th2 effector cytokines (100).Immunization with autologous tumor-derived GRP94 of mice bearing methylcholanthrene-induced fibrosarcomas effectively induced anti-tumor response on day 7 after tumor challenge and was less effective when the treatment was started on day 9 after tumor challenge (101). DCs primed with lung cancer-derived GRP94 also elicited anti-tumor response in cytotoxic T lymphocytes (CTL) and NK cells (102). On DCs, GRP94 interacts with TLR-2 and TLR-4, leading to increased expression of CD86 and secretion of IL-12 and TNF-α (103, 104). Intriguingly, eGRP94 also promotes the expression of Foxp3, IL-10 and TGF-β1 in T regulatory cells (T regs) *via* TLR2/TLR4- mediated NF-kB signaling pathway activation (105). GRP94-peptide complex interaction with TLRs appears to be critical for the stimulation of cytotoxic T cell response (106). Additionally, GRP94 also activates NLRP3 inflammasome in APCs, leading to the IL-1β secretion (107). Taken together, eGRP94 possesses both pro- and anti-tumor functions. On the one hand, eGRP94 promotes angiogenesis and supports T reg suppressive function and, on the other hand, eGRP94 induces DC maturation and enhances CTL response.

Intriguingly, Chen and colleagues demonstrated that expression of stress-inducible HSP90α on the surface of tumor-cell released autophagosomes (TRAPs) promotes IL-6 production by CD4+ T cells *via* TLR2-Myeloid differentiation primary response protein 88 (MyD88)- NF-kB signalling pathway (108). Autocrine IL-6 further enhanced IL-10 and IL-21 production by CD4+T cells *via* STAT3, supporting tumor growth and metastasis (108). In another study, eHSP90, IL-6 and IL-8 secreted by macrophages activated JAK2-STAT3 in pancreatic ductal epithelial cells, leading to malignant transformation of these cells (109). Recent data have emerged showing that monoclonal antibodies specifically targeting eHSP90α inhibited tumor formation *via* blocking the interaction of eHSP90 with matrix metalloproteinase 2 (MMP2) and MMP9 (110, 111). Since eHSP90α has a profound immunosuppressive effects, it may be further speculated that specific blocking of eHSP90α by monoclonal antibodies will dampen IL-6-dependent inhibitory effects on CD4+T and CD8+T cell function while also blocking IL-10 production by T and B cells, however, this warrants further investigation (108).

### Extracellular HSP70

**Figure 1:**
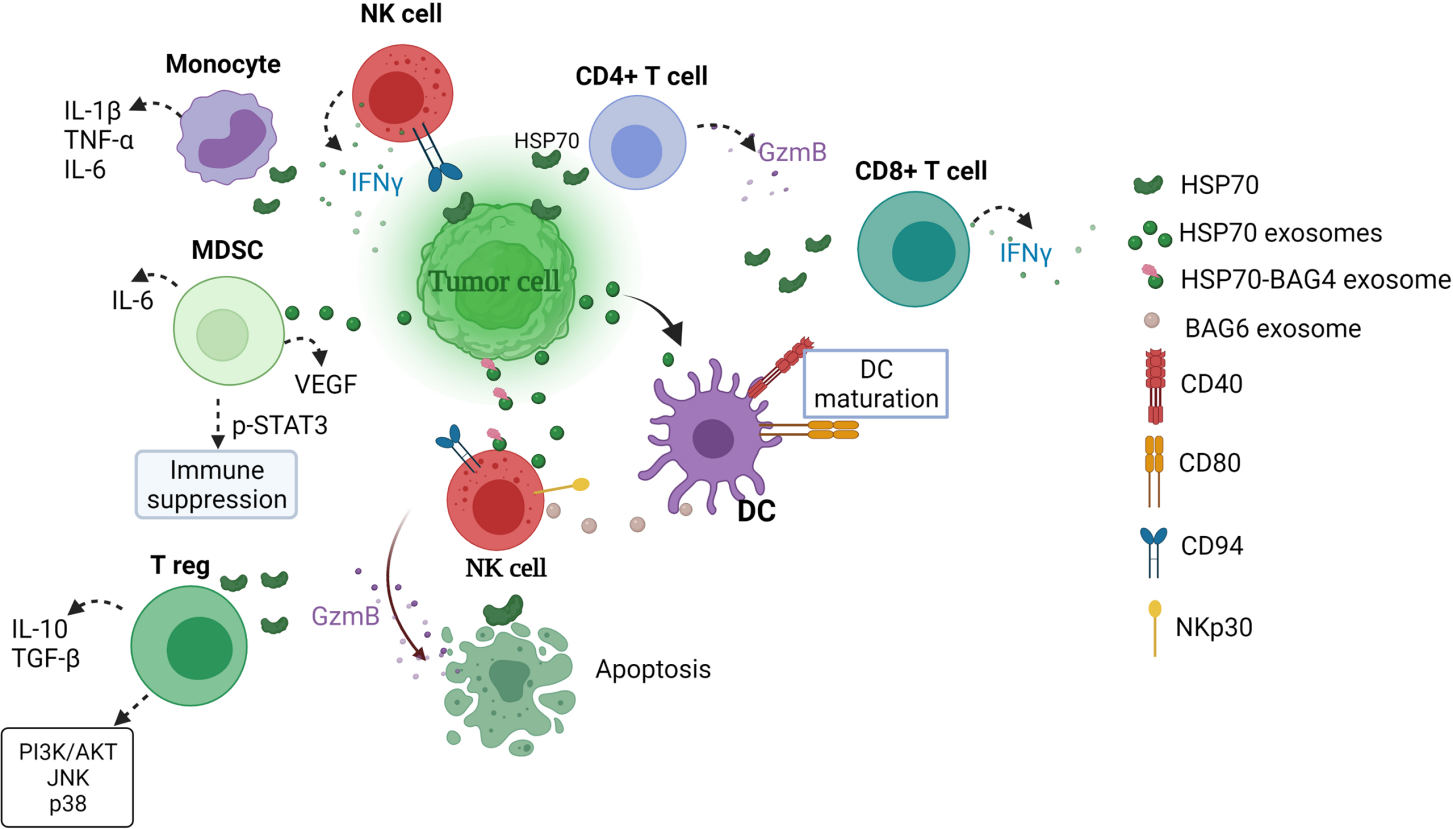
Extracellular HSP70 immune network in cancer. eHSP70s enhance NK cytotoxicity, DC maturation, induce strong CD4+ and CD8+ T cell responses and cytokine secretion by monocytes and enhance immunosuppressive activity of MDSCs and T regs (54, 112–118). MDSCs, myeloid-derived suppressor cells; T reg, T regulatory cell; GzmB, granzyme B; DC, dendritic cells; IFNγ, interferon γ; BAG, BCL2-associated athanogene; IL-6, interleukin-6; TNF-α, tumor necrosis factor α; VEGF, vascular endothelial growth factor; TGF-β, transforming growth factor β; PI3K/AKT, phosphatidylinositol 3-kinase (PI3K)-serine/threonine protein kinase (AKT), JNK, c-Jun N-terminal kinase; STAT3, signal transducer and activator of transcription.

eHSP70s have a dual role in the regulation of immune responses, where HSP70 can act as immune suppressor and immune activator (**Figure 1**). Such equivocal function of eHSP70 largely depends on the type of immune cell on which eHSP70 exerts its action. Multhoff and colleagues demonstrated that surface form of cytosolic stress-inducible HSP70 member acts as recognition structure for NK cells (119). Pre-treatment of NK cells with human recombinant HSP70 enhanced NK cell proliferation and IFN-γ production (54). Gastpar and co-workers have shown that CD94+NK cells migrate towards HSP70-surface positive tumors (112). Later, same research team demonstrated that the expression of HSP70 and co-chaperone BCL2-associated athanogene 4 (BAG4) on tumor-derived exosomes enhances migration and cytolytic activity of NK cells (113, 118). The expression of BAG6 on the surface of DCs-derived exosomes also activates NKp30 receptor (120). Following treatment with various chemotherapeutic agents, HSP-bearing exosomes released by hepatocellular carcinoma cells stimulates NK cell cytotoxicity and granzyme B production (121).

The ability of exosomal HSP70 to stimulate anti-tumor responses has been demonstrated with the use of engineered myeloma J558HSP cell line (115). Cells were manipulated to endogenously express P1A tumor antigen and membrane-bound HSP70 (115, 122). J558HSP –derived exosomes (EXO_HSP_) upregulated the expression of CD40 and CD80 and increased the section of IL-1β, IL-12, IFN-γ and TNF-α by DCs (115). Furthermore, immunization of BALB/c mice with EXO_HSP_ induced CD4+T cell response and secretion of IL-2 and IFN-γ, suggesting that EXO_HSP_ induces type 1 T helper response (115). EXO_HSP_ could also effectively elicit P1A-specific CD8+T cell response (115). In another study, stimulation of T cells with soluble HSP70 (sHSP70) increased IFN-γ, IL-6 and IL-8 production by CD4+ and IFN-γ and IL-8 by CD8+ cells (114). Notably, pre-stimulation with both HSP70 in combination with IL-2 increased granzyme B secretion by CD4+ and CD8+ T cells (114). Earlier, Blachere and colleagues demonstrated that GRP94/gp96-peptide complexes and HSP70-peptide complexes induce CD8+ T cell response (45).

eHSP70 also regulates immunosuppressive activity of myeloid-derived suppressor cells (MDSCs) and T regulatory cells (T regs) (116, 117). Exosomal HSP70 induces STAT3 phosphorylation and increases secretion of IL-6 and vascular endothelial growth factor (VEGF) in MDSCs, thus promoting tumor growth (116). Wachstein and co-workers demonstrated that pre-treatment of T regs with HSP70 upregulated the secretion of IL-10, transforming growth factor β (TGF-β) and resulted in phosphorylation of phosphatidylinositol 3-kinase (PI3K)-serine/threonine protein kinase (AKT), c-Jun N-terminal kinase (JNK) and p38 (117).

HSP70 ER homolog GRP78/BiP has also been observed on the surface of tumor cells (123). High GRP78 expression has also been observed on the surface of PBMC subpopulations including CD4+ and CD8+T cells and CD56+ NK cells, following chemotherapy in breast cancer patients (124). Interestingly, eGRP78 increases CD19+ surface expression, upregulates PD-L1 and FasL expression and IL-10 secretion in B cells (125). Notably, CD19+ cells pre-treated with GRP78 and anti-CD40 inhibited the proliferation of CD3/CD28- activated T cells, indicating that GRP78 may induce B regulatory cells (125). In another study, Corrigall and colleagues have reported that eGRP78 increased IL-10 secretion by PBMCs and reduced expression of HLA-DR and CD86 on monocytes (126). Later, same research team showed that mDCs treated with GRP78 increases intracellular indoleamine 2,3- dioxygenase (IDO) level and surface expression of leukocyte immunoglobulin-like receptor subfamily B member 1 and downregulates HLA-DR and CD86 expression, while retaining CD14 expression (127). T cells treated with such mDCs upregulated the expression of CD4^+^CD25^high^CD27^high^ and cytotoxic T-lymphocyte antigen (CTLA-4), while no increase in the expression of forkhead box P3 (FOXP3) was observed (127). Therapeutic targeting of surface GRP78 by human IgM monoclonal antibody PAT-SM6 resulted in induction of apoptosis and complement-dependent cytotoxicity in *de novo* and relapsed multiple myeloma (38, 128). Along this line, murine IgG antibody C107 targeting GRP78 carboxyl-terminal domain induced apoptosis *in vitro* and inhibited melanoma growth *in vivo* (123, 129). In another study, Liu and co-workers reported that monoclonal antibody Mab159 binds to the surface GRP78 and triggers GRP78 endocytosis, leading to apoptosis in breast and colon cancer cell lines *via* inhibiting phosphoinositide 3-kinase (PI3K) activity (130).

Pilzer and co-workers demonstrated that mitochondrial HSP70 mortalin interacts with complement C9 (131, 132). Sub-lytic complement attack causes C9 and mortalin release in extracellular vesicles (131). Mechanistically, formation of the complete C5b-C9 membrane-attack complex (MAC) induced the release of mortalin whereas targeting mortalin with antibodies showed to increase cell lysis (131). Thus, authors concluded that mortalin protects cells from complement-dependent cytotoxicity (CDC) by removing MAC from the cell surface (131). Later, same research team showed that blocking mortalin sensitizes tumor cells to CDC (133).

eHSP70 may also bind to the surface of human monocytes, leading to intracellular calcium flux, activation of nuclear factor (NF)-kB and increased production of IL-1β, TNF-α and IL-6 *via* NF-kB pathway (134).Taken together, extracellular HSP70s promote proliferation and cytolytic activity of NK cells, DC maturation, CD4+ and CD8+ T cell responses, protect cancer cell from CDC as well as enhances induction of tolerogenic DCs, immunosuppressive activity of MDSCs and generation of T regulatory cells. Therefore, future discoveries of therapies targeting extracellular form of HSP70 should take into account equivocal effect of HSP70 family members on different components of immune system.

### Extracellular HSP110 and GRP170

HSP110 represents a family of chaperones that is distantly related to HSP70 family (1). Recent studies have emphasized the role of extracellular HSP110 and its ER member GRP170 in the regulation of immune responses. In this regard, Berthenet and colleagues reported that eHSP110 promotes macrophage polarization towards M2 phenotype *via* TLR4 pathway while HSP110 inhibition reverses this effect (135). In the extracellular space, GRP170 secreted by B16 melanoma cells acts as a danger signal, inducing the production of IL-1β and TNFα by DCs and eliciting antigen-specific CTL response by cross-priming (136–138). Along this line, immunization of mice with tumor-derived GRP170 induces potent CD8+T cell response (139).

### Extracellular HSP60

**Figure 2:**
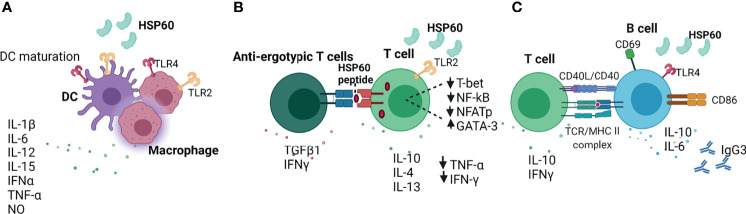
Extracellular HSP60 immune network. **(A)** HSP60 induces DC maturation and secretion of inflammatory cytokines (140–142). **(B)** eHSP60 downregulates Th1-associated transcription factors (T-bet, NF-kB, NFATp) and upregulates GATA3, leading to decreased secretion of TNF-α and IFN-γ and increased secretion of IL-10, IL-4, IL-13 (143). Activated T cells can present HSP60 *via* MHC molecules to anti-ergotypic T cells, leading to the production of IFNγ and TGFβ1 by anti-ergotypic T regulatory cells (144). **(C)** HSP60 activates B cells *via* TLR4-MyD88 signaling pathway, leading to the production of IL-10, IL-6 and IgG3 (145). TLR4, Toll-like receptor 4; NO, nitric oxide, CD40L; CD40 ligand; TCR, T cell receptor; MHC II, major histocompatibility complex; IgG3, Immunoglobulin G3; Nf-kB; nuclear factor kappa B; NFAT, nuclear factor of activated T cells.

HSP60 plays critical role in the regulation of innate and adaptive immune responses (**Figure 2**) (141). In response to HSP60, macrophages and DCs secrete inflammatory cytokines such as IFNα, TNF-α, IL-12, IL-15, IL-6, IL-1β and NO (**Figure 2A**) (141, 142). eHSP60 induces the maturation of bone marrow-derived dendritic cells (BMDCs) *via* TLR4 and activation of allogeneic T cells, resulting in the production of Th1-promoting cytokines (140). Feng and colleagues demonstrated that the expression of HSP60 on the surface of apoptotic tumor cells activates DCs and induces cytotoxic T cell response, suggesting that the HSP60 on tumor cells may promote potent anti-tumor T cell response mediated by APC (2, 146). By contrast, T cells pre-treated with HSP60 downregulate Th1-associated transcription factors such as T-bet, NFATp and NF-kB, inhibiting the secretion of IFN-γ and TNF-β, and upregulate GATA-3, leading to increased secretion of Th2-associates cytokines such as IL-10, IL-4 and IL-13 (**Figure 2B**) (143). eHSP60 also increases the expression of suppressor of cytokine signalling 3 (SOCS3) *via* TLR2 and STAT3, thus inhibiting T cell chemotaxis towards stromal cell-derived factor-1α (SDF-1α) (147). Activated T cells can also present HSP60 by MHC molecules to anti-ergotypic T regulatory cells, resulting in the secretion of IFN-γ and TGFβ1 by anti-ergotypic T cells (**Figure 2B**) (144). Of note, co-stimulation in the form of CD80, CD86 and CD28 is required for the activation of anti-ergotypic T cells (144). Additionally, anti-ergotypic T regulatory cells decrease the secretion of IFNγ by effector T cells *in vitro* (141, 144). eHSP60 also stimulates the secretion of IL-10, IL-6, IgG3 and upregulates the expression of MHC class II, CD69, CD86 and CD40 in B cells (145). Interaction of eHSP60-treated B cell with T cells leads to the IFNγ and IL-10 production by T cells (**Figure 2C**) (145).

In the extracellular milieu, HSP60 released by B16 melanoma cells promotes the secretion of immunosuppressive cytokines and chemokines including IL-6, IL-10, IL-13, TGF-β1, CCL-2 and CCR8 *via* TLR2 and STAT3 activation (2, 148). Highly metastatic B16 cells released higher levels of HSP60 resulting in persistent TLR2 and STAT3 activation compared to poorly metastatic B16-F1 cells (148). These results provide a mechanistic explanation to the role that extracellular HSP60s play in promoting immunosuppressive tumor microenvironment (2).

### Extracellular HSP27

HSP27 (HSPB1) is a member of small HSP family aberrant expression of which correlates with poor prognosis and resistance to chemotherapy in different types of cancer (1, 149). eHSP27 induces the secretion of immunosuppressive factors including IL-6, IL-10, prostaglandin E2 and proangiogenic cytokines such as IL-8, VEGF-A, IL-1β and TNF-α by human monocytes (150). eHSP27 also induces high level of monocyte chemotactic protein-1 (MCP-1), a chemokine responsible for monocyte recruitment at the tumor sites (150). Moreover, eHSP27 promotes the differentiation of monocytes into macrophages with TAM-like phenotype (150). HSP27-differentiated macrophages have reduced expression of MHC class II, CD86 and increased expression of PD-L1, Ig-like transcript 2 (ILT2) and ILT4 (150). Autologous T cell co-cultured with HSP27-differentiated macrophages inhibits T cell proliferation and significantly reduces the secretion of IFN-γ and IL-13 by T cells, suggesting that HSP27-differentiated macrophages induce T cell anergy (150).

## Conclusion and Perspectives

Heat shock proteins are molecular chaperones which have shown to be implicated in various hallmarks of cancer such as apoptosis resistance, angiogenesis, invasion, metastasis, cancer cell stemness and immune tolerance. Apart from their intracellular functions, HSP can also be secreted in extracellular space, where HSPs interact with various components of the immune system. Even though considerable progress has been made in deciphering the role of HSPs in tumor immunity, there is still a lot to be understood. For example, the role of distinct HSP members in the regulation of innate and adaptive immune responses inside and outside the cell in the context of cancer is not clear. Furthermore, the effects of various HSP-based immunotherapies on the release of HSPs in tumor microenvironment and their subsequent effects on immune responses are not yet fully understood. Taking into account that inside the cell HSPs may translocate from their primary locations and acquire different functions, it is also important to understand the effect of HSP-based immunotherapies on intracellular HSPs. Elucidating the role of HSP in the modulation of immune responses may improve current treatment strategies and open new perspectives for the discovery of novel HSP-based immunotherapy approaches.

## Author Contributions

ZA: conceptualization and manuscript writing. YM: administrational support. All authors contributed to the article and approved the submitted version.

## Funding

This research was funded by RFBR, project number 20-315-90081.

## Conflict of Interest

The authors declare that the research was conducted in the absence of any commercial or financial relationships that could be construed as a potential conflict of interest.

## Publisher’s Note

All claims expressed in this article are solely those of the authors and do not necessarily represent those of their affiliated organizations, or those of the publisher, the editors and the reviewers. Any product that may be evaluated in this article, or claim that may be made by its manufacturer, is not guaranteed or endorsed by the publisher.
